# Supplementary material for: Endosymbiotic Bacterial Diversity of Corn Leaf Aphid, Rhopalosiphum maidis Fitch (Hemiptera: Aphididae) Associated with Maize Management Systems
Source: Microorganisms. 2022 Apr 30;10(5):939. doi: 10.3390/microorganisms10050939 (PMC9145372; doi:10.3390/microorganisms10050939)
Supplement: Supplementary file 1 [file microorganisms-10-00939-s001.zip › microorganisms-1664363-supplementary.pdf]

Table S1. Supplementary online materials. Total bacterial taxa associated with maize fields and *R. maidis. Values represents DNA sequence numbers.*

Taxon	A01	A02	A03	A04	A05	A06	A07	A08	A09	A10	A11	A12	A13	A14
Acidicapsa	0	0	0	0	0	0	0	0	0	0	0	19	0	0
Edaphobacter	0	0	0	0	0	0	0	0	0	0	0	17	0	0
Bryobacter	0	0	0	0	0	2	0	0	0	0	0	20	2	0
Candidatus_Solibacter	0	0	0	0	0	0	0	0	0	0	0	6	0	0
Blastocatellaceae_unclassified	0	0	0	2	0	3	0	0	0	0	0	0	0	0
Subgroup	0	0	0	0	0	2	0	0	0	0	0	0	0	0
Vicinamibacteraceae	0	0	0	0	0	2	0	0	0	0	0	48	0	0
Actinobacteria	0	0	0	0	0	0	0	0	0	1	0	1	0	1
Corynebacterium	0	0	0	0	0	0	0	0	0	0	0	12	7	0
Lawsonella	0	3	4	4	0	0	0	0	0	0	0	2	0	0
Mycobacterium	0	0	0	0	0	4	0	0	0	0	0	33	0	0
Rhodococcus	0	0	0	0	0	0	0	0	0	0	0	27	0	4
Smaragdicoccus	0	0	0	0	0	0	0	0	0	0	0	3	0	0
Jatrophihabitans	0	0	0	0	0	0	0	0	0	0	0	11	0	0
Frankiales_unclassified	0	0	0	0	0	0	0	0	0	0	0	21	0	0
Geodermatophilaceae_unclassified	0	0	3	0	0	0	0	0	0	0	0	0	0	0
Sporichthyaceae_ge	0	0	0	0	0	0	4	0	4	1	0	3	11	0
Sporichthyaceae_unclassified	0	0	0	0	0	0	22	3	6	1	0	13	17	7
Quadrisphaera	0	0	0	0	0	0	0	0	0	4	0	0	0	0
Intrasporangiaceae_unclassified	1	0	5	0	0	0	2	0	0	0	0	4	0	0
Jonesia	0	0	0	0	0	0	0	0	9	0	0	0	0	0
Candidatus_Aquiluna	4	2	1	2	3	0	11	1	10	2	0	11	1	0
Curtobacterium	0	0	0	0	0	0	0	0	0	0	0	0	0	4
Microbacteriaceae_unclassified	28	6	33	16	15	4	21	0	35	5	2	462	0	18
Microbacterium	37	4	0	56	0	0	9	0	78	92	3	0	0	5
Rathayibacter	0	0	0	0	0	0	0	0	0	16	0	0	0	0
Subtercola	0	0	0	0	0	0	0	0	0	0	0	5	0	0
Kocuria	0	0	0	0	0	0	0	0	0	0	0	0	0	3
Micrococcus	0	1	0	0	2	0	0	0	10	0	0	0	0	0
Pseudarthrobacter	0	0	2	0	0	0	2	0	0	1	0	58	0	3

Micrococcales_unclassified	0	0	0	2	0	0	0	0	0	0	0	0	0	0
Micromonosporaceae_unclassified	0	0	0	3	0	0	0	0	0	0	0	5	0	0
Marmoricola	0	0	0	0	0	0	0	0	0	0	0	10	0	7
Nocardioides	0	0	0	7	0	9	0	0	0	0	0	26	0	3
Cutibacterium	0	2	0	0	0	0	0	0	0	0	0	0	0	0
Pseudonocardia	2	0	0	0	0	3	3	0	1	0	0	0	3	0
Actinobacteriota_unclassified	0	0	0	0	0	0	0	0	0	0	0	0	0	3
Rubrobacter	0	0	3	0	0	0	0	0	0	0	0	0	0	0
Gaiella	0	0	0	0	0	2	7	0	0	0	0	15	0	0
Gaiellales_ge	0	0	0	0	0	0	0	0	3	0	0	0	0	0
Gaiellales_unclassified	0	0	0	0	0	0	0	0	0	0	0	37	0	0
Conexibacter	0	0	0	0	0	0	0	0	0	0	0	3	2	0
Parviterribacter	0	0	0	3	0	0	0	0	0	0	0	0	0	0
Solirubrobacter	0	0	0	2	0	0	0	0	0	0	0	47	0	0
Solirubrobacterales_unclassified	0	0	3	0	0	0	0	0	0	0	0	0	0	0
Thermoleophilia_unclassified	0	0	0	0	0	0	0	2	0	0	0	26	0	0
Armatimonas	0	0	0	0	0	0	0	0	0	0	0	0	3	0
Armatimonadales_ge	0	0	0	0	0	0	0	0	0	0	0	3	0	0
Fimbriimonadaceae_ge	0	0	0	0	0	0	0	0	0	0	0	20	0	0
Fimbriimonas	0	0	0	0	0	0	0	0	0	0	0	3	0	0
Bacteria_unclassified	6	6	3	1	4	5	0	0	2	0	6	64	3	3
Bacteroides	0	0	3	0	0	0	0	0	0	0	0	0	0	0
Bacteroidales_unclassified	0	0	0	0	0	0	7	0	0	0	0	0	0	0
Dysgonomonas	0	0	0	0	0	4	0	0	0	0	0	0	0	0
Alloprevotella	0	0	9	0	0	0	0	0	0	0	0	0	0	0
Bacteroidia_unclassified	2	0	0	0	0	0	0	0	0	0	0	0	0	2
Aurantisolimonas	0	0	0	0	0	0	0	0	0	0	0	77	0	0
Chitinophaga	0	0	0	0	0	0	0	0	0	0	0	11	0	0
Chitinophagaceae_unclassified	0	0	3	0	0	0	0	0	8	0	0	208	0	0
Dinghuibacter	0	0	0	1	1	1	0	0	0	0	0	0	0	7
Edaphobaculum	0	0	1	0	0	0	0	3	3	0	0	48	17	0
Ferruginibacter	0	0	0	0	0	0	0	0	0	0	1	171	0	0
Flavisolibacter	0	0	0	0	0	0	0	0	0	0	0	0	4	0
Flavitalea	0	0	0	0	0	0	0	0	0	0	0	18	0	0
Heliimonas	0	0	0	0	0	0	0	0	0	0	0	6	0	0

Parafilimonas	0	0	0	0	0	0	0	0	0	0	0	2	0	0
Rurimicrobium	0	0	0	0	0	0	0	0	6	2	0	0	0	0
Sediminibacterium	0	11	1	0	0	1	1	2	1	1	5	298	5	0
Taibaiella	7	0	5	0	0	0	5	0	7	0	0	9	4	0
Terrimonas	0	0	0	0	0	0	0	0	0	0	2	73	0	0
Chitinophagales_unclassified	0	0	0	0	0	5	0	0	0	0	0	0	0	0
Cyclobacteriaceae_unclassified	0	0	0	0	0	0	0	0	0	0	0	5	0	0
Cytophaga	0	0	0	0	0	0	0	0	0	0	5	49	0	0
Siphonobacter	8	0	3	0	25	0	0	0	825	32	0	0	0	0
Adhaeribacter	0	0	0	0	0	0	0	0	0	0	0	30	0	0
Hymenobacter	0	0	2	0	0	2	0	0	0	0	0	47	0	0
Microscillaceae_unclassified	0	0	0	0	0	0	0	0	0	0	0	341	0	0
Arcicella	0	0	0	0	0	0	0	0	0	407	0	0	0	0
Dyadobacter	15	0	0	8	0	0	59	0	0	0	0	108	0	0
Emticicia	0	0	0	0	0	0	0	0	0	0	0	30	0	0
Larkinella	0	0	3	0	0	0	0	0	0	0	0	7	0	0
Pseudarcicella	0	0	2	1	0	0	5	10	0	4	0	0	1	6
Runella	1	0	0	0	0	1	0	0	0	0	0	0	6	0
Spiroplasma	0	0	0	0	0	0	0	0	4	0	0	47	0	4
Fluviicola	0	0	0	0	0	0	0	0	0	0	0	0	3	0
Flavobacterium	3	6	0	1212	0	7	0	2	1	3	0	277	12	5
Myroides	0	0	0	0	65	0	4	0	0	0	0	0	0	0
Chryseobacterium	7703	654	478	5476	1941	120	266	358	15355	8994	3	1	0	5368
Moheibacter	0	0	0	0	4	0	0	0	0	0	0	0	0	0
Mucilaginibacter	0	0	0	0	0	0	17	10	0	0	0	406	0	0
Pedobacter	15	0	130	7851	340	3	1201	16	1090	2375	0	32	0	0
Sphingobacteriaceae_unclassified	0	0	1	4	4	1	1	0	0	104	5	15	21	1
Sphingobacterium	25	0	0	10	3	3	0	0	0	0	0	11	0	0
Sphingobacteriales_unclassified	0	0	0	0	0	0	0	0	0	0	0	0	3	0
Bacteriovorax	0	0	0	0	0	0	0	0	0	0	0	12	0	0
Peredibacter	0	0	3	0	10	0	0	0	128	3	0	29	0	0
Bdellovibrio	0	0	0	3	0	0	0	0	110	0	0	151	0	0
Silvanigrellaceae_unclassified	0	0	0	0	0	0	0	0	0	0	0	0	4	0
Chloroflexi_unclassified	0	0	0	0	0	0	0	0	0	0	0	34	0	0
Chloroflexaceae_unclassified	0	0	0	2	0	0	0	0	0	0	0	0	0	0

Cyanobium_PCC-6307	0	0	0	0	0	0	8	0	0	0	0	0	0	0
Synechococcus_MBIC10613	0	0	3	0	0	0	0	0	0	0	0	0	0	0
Deinococcus	0	0	0	0	0	0	0	0	0	0	0	0	0	3
Anoxybacillus	0	0	0	0	0	0	0	2	1	0	0	9	0	0
Bacillales_unclassified	0	0	6	0	0	0	0	0	0	0	0	14	0	0
Paenisporosarcina	0	0	0	0	0	0	0	0	0	0	0	0	0	2
Exiguobacterium	0	3	0	0	0	0	0	0	0	8	0	32	0	3
Atopostipes	0	0	0	0	0	0	0	0	5	0	0	0	0	0
Granulicatella	0	0	0	0	3	0	0	0	0	0	0	0	0	0
Lactococcus	0	0	0	0	0	5	11	0	0	0	0	0	0	0
Streptococcus	0	0	13	0	0	0	0	0	0	0	0	33	0	0
Paenibacillus	0	0	0	0	0	0	0	0	5	0	0	0	0	0
Staphylococcus	0	0	0	0	0	0	10	0	0	0	0	18	0	0
Lachnoclostridium	0	0	5	0	0	0	0	0	0	0	0	0	0	0
Peptoclostridium	0	0	0	0	0	0	0	0	0	0	0	4	0	0
Anaerococcus	2	0	0	0	0	0	0	0	0	0	0	0	0	0
Fenollaria	0	0	0	0	0	0	0	0	0	0	2	0	0	0
Selenomonadaceae_unclassified	0	0	0	0	0	0	0	0	0	0	0	0	0	3
Thermoanaerobacterium	0	0	0	0	0	0	0	0	2	0	3	0	0	0
Fusobacterium	0	0	10	0	0	0	0	0	0	0	0	0	0	0
Gemmatimonadaceae_unclassified	0	0	0	0	0	0	0	0	0	0	0	22	0	0
Gemmatimonas	0	0	5	0	0	39	0	0	0	0	0	11	0	5
Haliangium	0	0	0	0	0	2	0	0	0	0	0	27	0	0
Aetherobacter	0	0	0	0	0	0	0	0	0	0	0	29	0	0
Pajaroellobacter	0	0	0	0	0	2	0	0	0	0	0	107	0	0
Polyangiales_unclassified	0	0	2	0	0	0	0	0	0	0	0	0	0	0
Nitrospira	0	0	0	0	0	0	0	0	0	0	0	0	4	0
Absconditabacteriales_(SR1)_ge	0	0	0	0	0	0	0	0	0	0	0	6	0	0
Candidatus_Kaiserbacteria_ge	0	0	0	0	0	0	0	0	0	0	0	54	0	0
Candidatus_Nomurabacteria_ge	0	0	0	0	0	0	0	0	0	0	0	3	0	0
Parcubacteria_unclassified	0	0	0	0	0	0	0	0	0	0	0	5	0	0
Saccharimonadaceae_unclassified	0	0	45	0	12	0	0	0	8	0	0	63	0	0
Saccharimonadales_ge	0	0	1	0	0	0	0	0	0	0	0	222	0	0
Saccharimonadales_unclassified	0	0	0	0	0	0	0	0	0	0	0	267	0	0
Phycisphaeraceae_unclassified	0	0	0	2	0	0	0	0	0	0	0	0	0	0

Tepidisphaera	0	0	0	0	0	0	0	0	0	0	2	89	0	0
Fimbriiglobus	0	0	0	0	0	0	0	0	0	0	0	4	0	0
Gemmata	0	0	2	0	0	0	0	0	0	0	0	49	0	0
Singulisphaera	0	0	0	0	0	2	0	0	0	0	0	0	0	0
Pirellula	0	3	0	2	0	4	0	0	0	0	0	0	17	3
Acetobacteraceae_unclassified	0	0	0	0	0	9	0	0	0	0	0	0	0	0
Acidiphilium	0	0	3	0	0	0	0	0	0	0	0	0	0	0
Gluconobacter	0	0	0	0	0	0	0	0	0	0	0	79	20	0
Roseococcus	0	0	0	3	0	0	0	0	0	0	0	3	0	0
Roseomonas	18	1	0	2	0	0	8	0	8	9	0	0	3	0
Alphaproteobacteria_unclassified	2	0	6	4	0	2	0	0	6	17	0	126	4	0
Azospirillum	0	0	0	0	0	0	0	16	0	541	0	0	0	0
Skermanella	0	0	0	0	0	0	4	0	0	0	0	0	0	0
Inquilinus	0	0	0	0	0	0	0	0	0	0	0	37	0	0
Asticcacaulis	0	0	0	0	0	0	0	0	0	0	0	12	0	0
Brevundimonas	0	0	0	0	6	3	25	0	0	5	0	27	0	20
Caulobacter	0	0	0	3	0	0	0	0	0	0	0	66	0	0
Caulobacteraceae_unclassified	0	0	5	0	0	3	0	0	3	0	0	3	0	0
Phenylobacterium	0	0	0	0	0	0	0	0	0	0	0	20	0	0
Hyphomonas	0	0	0	0	0	0	3	0	0	0	0	0	0	0
Dongia	0	0	0	0	0	9	0	0	0	0	0	6	0	1
Candidatus_Paracaedibacter	0	0	0	0	0	0	0	0	5	0	0	0	0	0
Reyranella	0	0	0	0	0	6	0	0	0	0	0	10	0	0
Beijerinckiaceae_unclassified	0	0	0	0	0	0	0	0	0	1	0	0	1	0
Bosea	0	0	0	0	0	0	1	1	0	0	0	0	0	0
Methylobacterium-Methylorubrum	0	0	73	2	0	1	0	8	8	15	0	60	0	0
Microvirga	0	0	0	2	0	0	0	0	0	0	0	0	0	0
Devosia	0	4	1	0	1	2	3	0	0	7	0	105	0	0
Devosiaceae_unclassified	0	0	0	0	0	0	0	0	0	0	0	56	0	0
Hyphomicrobium	0	0	0	0	0	0	0	0	0	0	0	12	0	0
Kaistia	0	0	0	0	0	0	2	0	0	0	0	65	0	0
Aureimonas	8	8	48	43	8	3	13	0	65	3745	1	14	0	14
Mesorhizobium	0	0	0	0	0	0	0	0	0	0	0	11	0	0
Ochrobactrum	0	0	0	0	1049	0	0	0	0	0	0	0	1	0
Rhizobiaceae_unclassified	4	0	0	19	15	0	0	0	2	0	0	0	0	0

Rhizobiales_unclassified	0	0	0	0	4	0	0	0	0	0	0	18	0	0
Bradyrhizobium	0	4	0	0	0	34	3	0	1	6	0	108	0	4
Pseudorhodoplanes	0	2	0	0	0	6	0	0	0	0	0	12	0	0
Rhodoplanes	0	0	0	0	0	0	0	0	0	0	0	25	0	0
Tardiphaga	0	0	0	0	0	0	0	0	0	0	2	228	0	0
Xanthobacteraceae_unclassified	0	0	0	0	3	2	0	0	0	0	0	16	0	0
Gemmobacter	0	0	0	0	0	0	6	0	0	0	0	0	2	0
Paracoccus	0	0	3	0	0	0	0	0	0	0	0	0	0	0
Rhodobacter	0	0	0	0	0	0	0	3	10	8	0	7	6	0
Rhodobacteraceae_unclassified	0	0	6	0	0	0	2	0	0	0	0	0	6	0
Tabrizicola	0	0	0	0	0	0	0	0	0	0	3	0	0	0
Wolbachia	0	0	94	14	0	0	0	0	0	0	16586	22362	0	0
Rickettsiales_unclassified	2	0	0	0	0	0	0	0	0	0	0	0	0	0
Altererythrobacter	0	0	0	0	0	0	0	0	0	0	0	65	0	4
Novosphingobium	19	0	0	5	1	0	11	0	0	0	0	88	0	1
Porphyrobacter	0	0	0	0	0	0	0	0	0	0	0	0	5	0
Rhizorhapis	0	0	0	0	0	0	2	0	0	0	0	0	0	0
Sandarakinorhabdus	0	0	1	0	0	1	3	0	1	0	0	0	9	3
Sphingobium	0	0	0	394	0	0	0	0	0	0	0	57	3	0
Sphingomonadaceae_unclassified	0	0	0	0	0	0	2	0	23	2	0	24	0	3
Sphingomonas	306	153	981	1028	577	63	541	88	7501	10840	18	764	6	420
Sphingorhabdus	0	0	0	1	1	0	0	0	0	0	0	1	0	0
Rheinheimera	0	0	0	0	0	0	2	0	0	0	0	0	0	0
Pseudoalteromonas	0	0	0	0	0	0	0	0	0	0	0	18	0	0
Pigmentiphaga	3	0	0	3	0	0	0	0	0	0	0	0	0	0
Verticiella	309	3	0	202	39	0	0	3	224	6	0	0	0	0
Burkholderiaceae_unclassified	0	0	0	0	0	0	2	0	0	0	0	0	0	0
Polynucleobacter	11	4	6	7	7	5	29	11	1	17	11	14	51	23
Ralstonia	0	0	0	0	0	0	0	0	0	0	0	51	0	0
Burkholderiales_unclassified	2	0	0	0	0	2	0	0	0	0	0	14	2	0
Acidovorax	0	0	0	0	0	0	0	0	14	0	0	0	0	0
Caenimonas	0	0	0	0	0	0	0	0	0	0	0	45	0	0
Comamonadaceae_unclassified	1662	6	174	1271	94	0	3	27	1440	47	0	108	0	4
Comamonas	483	0	0	0	0	0	0	0	0	0	0	0	0	0
Delftia	0	0	0	0	0	0	0	0	438	2	0	0	0	0

Lampropedia	0	0	0	0	26	0	0	0	0	0	0	0	0	0
Leptothrix	0	0	0	0	0	0	0	0	0	0	0	14	0	0
Limnohabitans	2	0	0	1	0	0	4	1	1	1	0	1	26	17
Paucibacter	0	0	0	0	0	0	0	0	0	0	0	11	0	0
Ramlibacter	0	0	0	0	0	0	0	0	0	0	0	11	0	0
Rhizobacter	0	0	0	0	0	0	0	0	0	0	0	19	0	0
Rhodoferax	0	0	0	0	0	0	0	0	0	0	0	0	2	0
Sphaerotilus	0	0	0	0	0	2	0	0	2	0	0	1	0	0
Variovorax	15	0	0	71	11	0	515	0	16	23	0	66	0	0
Hydrogenophilus	0	0	4	0	0	0	0	0	0	0	0	0	2	0
Methylophilaceae_unclassified	0	0	0	0	0	1	6	0	0	0	0	530	0	1
Methylotenera	0	2	5	0	0	0	0	0	0	0	0	2	0	0
Neisseriaceae_unclassified	0	0	0	0	0	0	2	0	0	0	0	0	0	0
Ellin6067	0	0	0	0	0	7	0	0	0	0	0	0	0	0
Duganella	12	4	95	2481	17	0	280	14	32	1298	0	1	6	7
Herbaspirillum	0	0	1	69	1	0	2	7	7	2400	1	0	0	0
Massilia	0	0	0	0	0	3	0	0	0	0	0	48	0	4
Oxalobacteraceae_ge	0	0	0	0	0	0	0	0	0	0	0	2	0	0
Oxalobacteraceae_unclassified	0	0	0	0	0	0	0	0	0	3	0	7	0	0
Cellvibrio	0	0	0	0	0	0	0	0	0	0	0	32	0	0
Coxiella	0	0	0	0	0	0	0	0	0	0	0	6	0	0
Gibbsiella	0	0	0	3	0	0	0	0	0	0	0	0	0	0
Enterobacterales_unclassified	13	7	0	32	86	16	365	0	0	0	48	2	0	2
Enterobacteriaceae_unclassified	10	0	0	0	0	0	0	0	0	0	0	0	0	0
Raoultella	942	0	0	0	27	0	0	0	0	0	0	0	0	0
Erwinia	5762	877	0	5805	15599	5	78470	117	11	56	0	0	23	1664
Erwiniaceae_unclassified	2	0	0	47	49	0	226	0	0	0	0	0	0	0
Rosenbergiella	0	0	0	0	0	0	13	0	0	0	0	4	1117	4
Buchnera	1852	77628	77706	3778	10808	72051	63720	70886	24704	18610	12136	62604	122689	114848
Candidatus_Hamiltonella	0	0	0	0	0	0	0	0	0	0	0	0	0	1969
Moellerella	0	0	4	0	0	10	0	0	0	0	0	0	0	0
Morganellaceae_unclassified	4	14506	15988	3	8	51330	5	1	0	0	7	0	0	34
Pectobacterium	0	0	4	0	0	0	0	11	0	0	0	0	0	0
Rahnella1	76	0	0	2	180	0	11	0	0	0	0	0	0	0
Serratia	6	1	41	0	0	0	31	0	0	2	100837	1689	3	83

Yersiniaceae_unclassified	13	0	0	3543	6	0	0	0	0	0	0	0	0	19
Acidibacter	0	0	0	0	0	0	3	0	0	0	0	17	0	0
Gammaproteobacteria_unclassified	0	0	0	6	4	2	0	0	0	0	8	0	0	0
Legionella	0	0	0	0	0	0	9	0	0	0	2	28	0	0
Alcanivorax	0	0	4	0	0	0	0	0	0	0	0	0	0	0
Halomonadaceae_unclassified	0	0	12	0	0	0	0	0	0	0	0	0	0	0
Acinetobacter	8814	0	0	1757	40	0	1	0	0	0	0	93	0	406
Enhydrobacter	0	0	0	0	0	0	0	1	0	55	0	0	0	0
Pseudomonadaceae_unclassified	2	0	0	24	23	0	0	6	2	9	0	0	0	0
Pseudomonas	19895	1364	2174	31416	54571	232	3373	23380	17637	23711	2	1851	64	656
Hydrocarboniphaga	0	0	0	0	0	0	0	0	0	0	0	6	0	0
Nevskia	0	0	0	0	0	0	0	0	0	0	0	0	2	0
Panacagrimonas	0	0	0	0	0	0	0	0	0	0	0	4	0	0
Vibrio	0	0	0	0	0	0	0	0	0	0	0	11	0	0
Dokdonella	0	0	0	0	0	1	0	1	0	0	0	0	0	0
Rhodanobacter	0	0	0	0	0	0	0	0	0	0	0	57	0	0
Arenimonas	0	0	0	0	0	0	0	0	0	0	0	44	0	0
Pseudoxanthomonas	0	0	0	0	0	0	0	0	0	0	0	12	0	0
Stenotrophomonas	2487	5	0	6	1376	0	2	17	0	0	0	21	0	27
Thermomonas	0	0	2	0	0	0	0	0	0	0	0	0	0	0
Xanthomonas	0	0	0	0	0	0	0	0	0	0	0	19	0	3
Neochlamydia	0	0	0	0	0	0	0	0	0	0	0	6	0	0
Parachlamydiaceae_unclassified	0	0	0	0	0	0	0	0	0	0	0	9	0	0
Candidatus_Udaeobacter	0	0	0	0	0	10	0	0	0	0	0	49	0	0
Chthoniobacter	0	0	0	2	0	0	0	2	0	0	0	40	0	0
Terrimicrobium	0	0	0	0	0	0	0	0	0	0	0	169	0	0
Candidatus_Xiphinematobacter	0	0	0	0	0	0	0	0	0	0	0	28	0	0
Opitutaceae_unclassified	0	0	0	0	0	0	0	0	0	0	0	43	0	0
Opitutus	0	0	0	0	0	0	0	0	0	0	0	31	0	0
Pedosphaeraceae_ge	0	0	0	0	0	3	0	0	2	0	0	6	0	0
Luteolibacter	0	0	0	0	0	1	5	0	0	1	4	114	10	4
Prosthecobacter	0	0	0	0	0	0	0	0	1	1	0	2	0	21
Verrucomicrobium	0	0	0	0	0	0	0	0	0	0	0	21	0	0
